# Expectations towards participation in easily accessible pain management interventions: a qualitative study

**DOI:** 10.1186/s12913-017-2668-3

**Published:** 2017-11-10

**Authors:** Torunn Hatlen Nøst, Aslak Steinsbekk, Liv Riseth, Ola Bratås, Kjersti Grønning

**Affiliations:** 1 Department of Public Health and Nursing, Norwegian University of Science and Technology, Postbox 8905, 7491 Trondheim, Norway; 2 Centre for Health Promotion Research, Norwegian University of Science and Technology, Trondheim, Norway; 3 3T- Fitness Center, Trondheim, Norway

**Keywords:** Chronic pain, Self-care, Expectation, Health services accessibility, Primary health care, Qualitative research

## Abstract

**Background:**

People with chronic pain use a range of healthcare services, but they also report a high degree of dissatisfaction with treatments. One reason for dissatisfaction might be participants’ expectations towards treatments. The aim of this study was to explore expectations of people with chronic pain towards participation in easily accessible pain management interventions delivered in public primary care.

**Methods:**

A qualitative study using semi-structured individual face-to-face interviews with 21 informants. The informants were recruited among participants enrolled in a randomised controlled trial on the effect of an easily accessible self-management course for people with chronic pain. The data were analysed thematically using Systematic Text Condensation.

**Results:**

Having experienced pain for a long time, there was no specific expectation of a cure or a significant alleviation of the pain. The informants’ expectations mainly concerned a hope that participation could lead to a better everyday life. The informants said that hope was important as it motivated them to keep going and continue self-care activities. The hope acted as a driving force towards trying new interventions and maintaining motivation to do activities they experienced as beneficial. Both concrete aspects of the current intervention and an understanding of what interventions in general could offer contributed to the informants hope. The expectations centred about the interventions being something new, as they had not previously tried this service, an opportunity to gain and reinforce skills, to help them continue to grow as a person, to meet others in similar situations, and to access professional support in an easy manner. Participating in interventions provided by healthcare services was seen by some as an act of self-care, where they did something active to manage their health.

**Conclusions:**

Expectations towards the interventions were related to a hope for participation leading to a better everyday life. The role of hope for peoples’ motivation to self-care implies that service providers should be aware of and help to maintain hope for a better everyday life. The importance of social support as part of self-care should be acknowledged when developing interventions targeting chronic pain.

**Trial registration:**

ClinicalTrials.gov: NCT02531282. Registered on August 21 2015.

## Background

Chronic non-malignant pain is a long-term condition estimated to affect approximately 19% of the adult European population [1]. Chronic pain is different from acute pain as it persists when treatment stops [2]. The long-term aspect of chronic pain challenges society with socioeconomic consequences such as early retirement, disability pensions, increased sick leave and healthcare utilization [3–5]. For individuals, its impact has been investigated in several studies, providing insight into physical, social and psychological consequences such as poor quality of life [4, 6], sleep disturbances [1, 5], exhaustion [5, 7], mood disturbances [8, 9], and interference with recreational activities and family responsibilities [4, 10].

Self-care is highlighted as important when managing long-term conditions [11], covering the actions people take to engage in behaviours that improve their health and wellbeing [12, 13]. Among people with chronic pain, typical self-care activities comprise physical activity and exercise [14, 15], alternating between strenuous and less strenuous activities [7, 16], and continuing everyday activities to bring structure and meaning to life [16]. Other self-care activities are aimed at distraction from pain by, for instance, listening to music [17], using heat to relieve the pain [7], and replacing thoughts leading to anxiety with more rational thoughts [18, 19].

The fact that total recovery often is not within reach [10, 20] makes coping with chronic pain a highly demanding and continuously ongoing task [20, 21]. There are a range of different interventions offered to and used by people with chronic pain [22], e.g., medication, surgery and nerve blocks, exercise and physical rehabilitation, psychological treatments, and complementary and alternative treatments. Thus, how people with chronic pain manage healthcare as part of their pain management is a central self-care activity. However, the effects of pain treatments are mostly reported as small to modest [22–24]. In addition, a high degree of dissatisfaction with pain treatments has been described [4, 25, 26].

Peoples’ expectations towards treatment are suggested as a possible reason for the dissatisfaction [22], indicating that expectations are important when considering how different types of interventions are experienced. Furthermore, a mismatch between patients’ needs and the delivery systems have been found [27], emphasizing the importance of knowing the participants’ expectations and aligning these with what the services offer. Expectations have been described as something one could expect or predict to happen, and also as normative or ideal expectations such as aspirations, hopes and desires [28, 29]. Thus, when people with chronic pain seek new treatment options, they are likely to have a range of expectations based on previous experiences with healthcare services [30]. Studies on patients’ expectations towards multidisciplinary treatments for pain found that participants expected to take an active part in the programmes and to learn adequate coping strategies to improve their daily life [13, 30]. Other studies on multidisciplinary and comprehensive interventions for chronic pain found that the participants expected to learn about diagnostics, pain causes, and to receive instructions and advice regarding their specific pain management [31, 32].

However, to the best of our knowledge, there is no publication on what people with chronic pain expect from participation in easily accessible pain management interventions. Thus, the aim of this study was to explore the expectations of people with chronic pain towards participation in easily accessible pain management interventions. The study was embedded in a randomised controlled trial (RCT) investigating the effect of an easily accessible self-management course for chronic pain in public primary care.

## Methods

This was a qualitative study with semi-structured individual face-to-face interviews. The interviews were conducted from September 2015 to April 2016.

### Setting

The study was embedded within a RCT investigating the effect of an easily accessible self-management course in public primary care for people with chronic pain. The protocol for the larger trial with a description of the intervention has been published previously [33]. In the RCT, participants were randomised to a chronic pain self-management course (intervention) or to a drop-in outdoor physical activity (control) [33]. Both activities were delivered at a Healthy Life Centre (HLC) in a city with approximately 190,000 inhabitants in Central Norway.

The HLC is a public service offered by Norwegian municipalities as part of their public healthcare service. The HLCs offer interventions with few barriers for participation and people can attend both with and without referrals from health professionals [34]. The HLCs deliver several group-based activities to support people in health behavioural changes and to manage chronic conditions, ranging from physical activity and exercise groups to smoke cessation programmes and coping with anhedonia courses. While participation in most interventions at the HLC is covered by Norway’s public health insurance, some have a small user fee of about USD 36/ EUR 34 for one course. The current HLC initiated the pain self-management course in line with non-disease-specific self-management interventions being transferred from hospitals to primary care. As such, the course aimed to be a supplement to follow-up pain sufferers receive from e.g., general practitioners, physiotherapy delivered in both public primary healthcare and private, and referrals to specialist care such as organ or disease-specific specialists and multidisciplinary pain clinics located at hospitals.

Participants were recruited to the RCT from general practitioners, physiotherapists, from advertisements in newspapers, websites, social media, and by email invitations to patient organisations. They were informed that the activities would be delivered in groups at daytime for a period of 6 weeks [33]. The participants received information in an information leaflet, in the informed consent and orally when they met for the baseline assessment. There was no user fee in the trial.

### Informants and recruitment

The inclusion criteria for the qualitative study was the same as for the RCT; adults of 18 years or older, self-reported pain for 3 months or more, and able to participate in group-discussions in Norwegian. Exclusion criteria comprised not being able to participate in easy physical activity for 1 hour (as in the activity offered the control group), pain arising from malignant diseases, and not having the capacity to consent and participate.

Informants to the qualitative study were recruited by inviting some of the participants enrolled in the RCT. The selection was mainly done by consecutively asking participants if they were able to meet for baseline assessment at specific time points, which was scheduled with extra time for the interviews, i.e., that they wanted to participate and had the time to be interviewed. By asking consecutively, we expected to get sufficient variation among the informants. All but one of those asked (did not have time for the interview), accepted and agreed to take part in the qualitative study.

Recruitment continued until 21 participants had been interviewed. At this point, we considered to have sufficient data to explore the research question in depth.

### Data collection and interview guide

The first author conducted all interviews, either at the Healthy Life Centre or in a meeting room at the research centre where the first author was located. The interviews were carried out before randomisation, i.e., before anyone knew whether they were allocated to the intervention or to the control group. Baseline questionnaires and tests of the RCT were completed before interviews to reduce the risk of reporting bias due to the interviews. The interviews lasted between 23 and 72 min (mean duration 43 min). Additional notes and reflections were written down immediately after each interview. To check if the interview guide needed alterations, the first and last author read the transcripts from the first three interviews. Minor changes were made in the sequence of questions but no new topics were added. No repeating interviews were conducted. The questionnaires completed at baseline for the RCT provided demographic data on the informants.

The interview guide was semi-structured with open-ended questions to allow the informants to speak freely. The topics were derived from the research question, literature, and the research group’s experience. The main interview question was: “*Can you tell about your expectations towards participation in the interventions*?” Follow-up questions were: “*Can you tell how you experience pain in your everyday life?*”, “*Can you tell about the activities you do to live as well as possible?*”, and finally, “*Can you tell about the healthcare services you have attended previously?*”. The interview proceeded as a conversation with the goal of exploring different aspects of the informants’ expectations towards what participation in the interventions could lead to.

### Data analysis

The interviews were audio recorded and transcribed verbatim. The data were analysed using Systematic Text Condensation (STC), a descriptive thematic cross-case analysis strategy based on a phenomenological approach [35]. STC was chosen as it is a structured and well described step-by-step method for analysis of qualitative data shown to be suited for presenting experiences as expressed by the informants rather than exploring possible and underlying meaning of their sayings [35].

The analysis followed the iterative four-step procedure of STC [35]. In the first step, the first author read all transcripts. The other authors read the same three transcripts chosen by the first author to be the ones with the most richness of data, to gain an overall impression of the data and to identify preliminary themes. These were discussed, resulting in seven preliminary themes associated with the informants’ previous experiences and current expectations. In the second step, the first author systematically reviewed the transcripts line by line to identify meaning units representing all parts of the interviews relevant for the research question. The meaning units were coded, classified and sorted into code groups related to the preliminary themes. These were discussed repeatedly in the author group and thereafter the preliminary themes were adjusted. In the third step, the first author performed a systematic abstraction of meaning units within each of the themes, reducing the content into a condensate that maintained the informants’ sayings. All authors read the condensates before another round of iterative discussions, resulting in several adjustments and renaming of the themes. In the final step, the content of the condensates was synthesised into generalised descriptions and concepts, while ensuring that the result still reflected the original context.

The first author identified illustrative citations which were discussed in the author group to choose the ones most illustrative. The citations were translated by the first author and validated by the co-authors. A person fluent in both Norwegian and English did a back translation from English to Norwegian to verify that the content was present in the translated citations. The citations used to support the results are marked with the informant’s gender, age group, and pain duration.

Analysis was data-driven with no theoretical framework as a template. The findings were repeatedly checked against transcripts for validation during the whole process and especially after the final analysis. MindManager [36] and NVivo 11.0 [37] were used as systematisation tools.

## Results

Twenty-one informants, 17 females and 4 males, aged 32–74 years (mean age 52 years), were interviewed (Table 1). Only two informants had heard of the HLC before and none knew this was a service that could provide support to manage long-term conditions.

**Table 1:** Demographic characteristics of the informants

Characteristics	Number
Gender	
Female	17
Male	4
Age (years)	
< 35	2
35–50	7
51–60	6
61<	6
Civil status	
Partner/ married	13
Divorced/ widowed/ single	8
Current work status	
Working	3
Sick leave	3
Disability pension	11
Retired	4
Pain duration (years)	
1–5	7
6–9	1
10 or more	13
Main reason for pain	
Osteoarthritis, rheumatic diseases, osteoporosis	9
Musculoskeletal pain, back pain, fibromyalgia	7
Neurological pain, migraine	3
Injuries after treatment, trauma	2

**Fig. 1:**
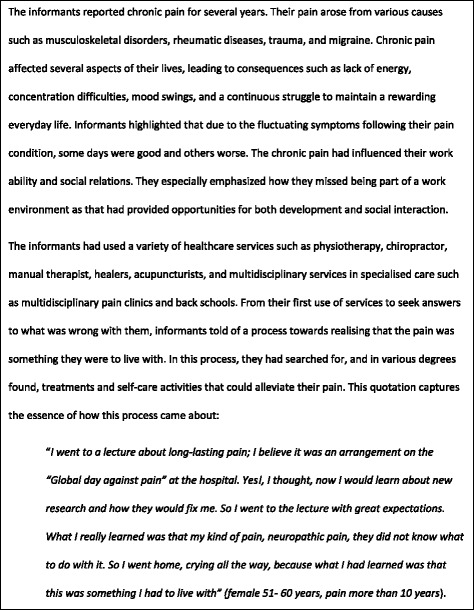
An overview of the informants’ previous experiences of living with chronic pain and the type of services they have used

The informants’ descriptions of living with chronic pain were similar to findings in other studies [7, 16, 17, 38], and therefore not elaborated on here. However, to give an impression of the informants’ previous experiences of pain and the health services they had used, we have added some information on the informants’ background in Fig. 1.

The authors’ overall understanding was that expectations towards participation in the easily accessible interventions were related to a hope that participation would lead to a better everyday life. This was a common overarching theme throughout and is presented as the first theme in the results: “Hope for a better everyday life”. Expectations in terms of what they hoped to experience were categorized into the following five sub-themes; “Something new and untried”, “To gain and reinforce skills”, “To continue to grow as a person”, “To meet others in similar situations”, and “To access professional support in an easy manner”.

### Hope for a better everyday life

Informants’ willingness towards participating in activities, trying new treatments and making changes in life was related to alleviating the pain and its consequences the best way they could. Most informants said they hoped that participation in the easily accessible interventions at the HLC would contribute to a better everyday life, using words like getting new insights, reinforcing skills, and meeting others who shared experiences of living with chronic pain. This was similar to what they said they hoped for when they had attended other services previously. Some informants emphasized that they saw it as important to carry the hope that life could get better despite their chronic condition, while at the same time acknowledging that the pain was likely to persist. It was said that hope was important as it motivated them to keep going and continue self-care activities.

*“I do not think I will be free from pain. But I do think I can manage it better. I hope it will be better. I believe that it can” (female, over 60 years, pain more than 10 years).*

The following sub-themes present the expectations towards the easily accessible interventions at the HLC and thus how they hoped participation could bring about a better everyday life.

### Something new and untried

Most informants spontaneously said they wanted to participate in the study because the HLC represented a new and untried service. They wanted to see if this service could add something new to their pain management. Some informants said the information of the content in the intervention focusing on how to think about pain was new to them as they had little knowledge on cognitive techniques for managing pain. Thus, they were curious about how to approach their thoughts on pain. One informant said that how to use his mind to manage pain was a mystery to him and therefore he wanted to participate. Another informant said it like this:

*“I just have to figure out how to think about something else, because sometimes, the pain just fills my body and my head so much that I cannot think. That is what I hope the course can give me. Those techniques to get my mind faster out of things” (female 35–50 years, pain more than 10 years).*

Some informants said they were were looking for input on how to alleviate pain without using medication. They experienced some drugs to be limiting as it kept them from, e.g., driving, and they were not comfortable being with grandchildren when they took drugs such as morphine. These informants especially stressed that they hoped to find other ways to alleviate pain as a reason for their participation. For some, the nature of the interventions indicated participation would not worsen their pain. One informant summed up her expectations on participation like this:

“*I have nothing to lose by participating. I do not believe it will be revolutionizing my life but I thought that this was yet untried. And suddenly, it could be something there that gives me; yes, maybe I will get a new insight*” *(female 35–50 years, pain duration 1–5 years).*

### To gain and reinforce skills and techniques

Several informants said it was important for them to do something actively to alleviate pain, and referred to how they tried to follow recommendations on nutrition and physical activity. However, some informants described that translating knowledge into practical action was challenging. One informant said she had knowledge on benefits of exercise related to her illness, but she was still looking for the best way to practice it. She hoped participation in a new activity could give her appropriate exercises, in addition to inspiration and a push to establish a routine for physical activity.

Some informants said that in the beginning of their illness they had participated in interventions, hoping and expecting the pain to go away. As their experiences with pain treatments increased, some said they changed preferences for what they looked for. One informant explained how she at first had tried anything to get rid of the pain, but now she had decided to look for activities to make her days as good as possible.

Another informant described that maintaining her health and functional level was a demanding and never-ending task. Therefore, from time to time, she needed interventions to refill her motivation and give her energy to keep using the skills she already had. Another informant had learned techniques at a pain clinic on how to think about something other than the pain. The techniques had faded with time and she therefore wanted to reinforce her skills and hoped that the new interventions would fulfil this need.

*“I feel that I have been at several health services so to speak, in relation to coping and all that. But of course, one forgets things after a while, and then you have to pick it up again” (female 35–50 years, pain 1–5 years).*

### To continue to grow as a person

Some informants hoped participation could contribute to releasing potential in themselves that they considered to be unexpressed. Thus, they wanted to participate in activities that could help them to grow and develop as a person. One informant said she perceived to have good knowledge on how to manage pain but at the same time, she thought it was possible for her to expand her understandings. However, she struggled to find interventions that provided input to bring her further in her pain management.

Others spoke of wanting to develop their skills and talents to reach as far as possible towards their goals in life. For some, this was about finding meaningful activities that challenged them and prevented stagnation. One informant emphasized the importance of learning new things at every opportunity.

*“Still, I believe I have potential. I mean I can do more, achieve more. I still see that I am capable of development in many ways. And then I think that if I do not improve myself or if I do not learn something new every day, then that belief will die” (male over 60 years, pain more than 10 years).*

For him, it was natural to seek activities like the intervention at the HLC, as he no longer perceived input and opportunities for development at work. Another informant said she wanted to participate because she needed to push herself outside her comfort zone. She looked for opportunities to develop herself since she no longer participated in work life.

### To meet others in similar situations

When talking about expectations towards participation, most informants immediately emphasized how important it was to be with other people. They said support from others helped them in their struggles and efforts to hold the pain at bay. Some informants said they had worn out people closest to them, and others described how their condition was difficult for others to understand. One informant expressed that she needed her condition to be recognized as challenging. This made her search for settings where her challenges would be acknowledged. One informant who hoped she would meet people who understood her situation, said:

*“It is the worst part of having long-term pain. That nobody cares. For no one can truly understand what you really are going through” (female 51–60 years, pain more than 10 years).*

For some informants, being on sick leave or disability pension had led to a lot of available time they wanted to fill with meaningful activities. One informant said she missed having something to do with other people, especially during the daytime. Others said they hoped the intervention could be a regular activity to attend. In addition, they hoped participation would be an opportunity to help others by sharing their own experiences, but also to learn by listening to other peoples’ experiences. One informant who had lived with pain for more than 10 years and had undergone several surgeries had never participated in interventions with others having similar health challenges. She said she was excited about the opportunity to hear how others managed to live with pain. For some, meeting others was also expected to give perspective to their own situations.

*“It would be nice to get some input on other ways to manage pain. To have the benefit of others experiences and advice, on how they manage things so to say” (female 51–60 years, pain more than 10 years).*

### To access professional support in an easy manner

Most informants said they were not concerned whether interventions were delivered at a hospital or in primary care. More important was that there actually was a service available when they needed help to manage the pain. That was something they hoped to have found when they were informed of the HLC concept. One informant said she experienced each attended healthcare service as an assembly line where she came in, was treated, got out and then there was nothing more. To know where to turn for further support was challenging and she was looking for a service she easily could access, even just as a place to call. Another informant summed up her interactions with health services like this:

*“In many ways I have missed that someone took care of me. Because no one cares about my health. My GP just writes out prescriptions, and then- was it something else? So, that is what I hope for really. To be taken care of. Because everyone needs that; to be seen” (female 51–60 years, pain more than 10 years).*

Some informants had experiences of referrals to services being declined because their condition did not fit the priorities of the service. Others said there were waiting lists for services they wanted to attend, and some found it difficult to manage the costs allocated to treatments. They appreciated the low cost for interventions at the HLC compared to other services they had tried. Some stated the easy access as central for their participation because it enabled them to take control over their healthcare. They said it was important that requests for help were appropriately met. Participating in interventions was described as a way of self-care where they did something active to manage their health. One informant summarised her views like this:

*“I believe it is important for society to take care of people so they can be in good shape for as long as possible. Self-care as long as possible. Even though they are not in paid work, I think it is really important. Because if you manage to get people with long-term pain in activity, then you will keep them healthy much longer” (female 51–60 years, pain more than 10 years).*

## Discussion

The expectations towards the easily accessible interventions were related to a hope that participation could lead to a better everyday life. As the HLC for most of the informants was an untried service, it represented a possibility for maybe finding something new that could make their life with pain a little bit easier. The new and untried intervention provided an opportunity to meet other people, to learn or reinforce skills, and to develop as human beings. This gave hope of maybe having found a service that would be easy to access whenever they needed support to manage their pain.

### Expectations that participation could lead to a better everyday life

The informants’ expectations were related to a hope for the possibility that despite their chronic condition, everyday life could become better. Consequently, they expressed a willingness to try anything that could contribute to minimising the pain’s interference with their everyday life regarding social activities, family responsibilities and participation in work life. Although there are differences in how the term hope is understood in studies among patients, comprising terms such as expectations, aspirations, wants and desires, [28, 29], informants in our study spoke about hope without making such differentiations. Nevertheless, hope has been pointed to as central in how people with pain assess new experiences and adjust their expectations towards treatments [39], and our findings support this.

The informants in our study believed, despite having experienced modest effect of treatments, that it was possible to alleviate the consequences of their pain. As such, our results are consistent with understanding chronic pain as a condition where the pain itself can become secondary to its consequences on everyday life [40]. For the pain level to be substantially reduced, however, the informants expressed few expectations. One reason might be that the information about the interventions content did not encourage expectations of pain relief as it focused more on what they could do themselves to make everyday life better. A recent review, however, found patients’ expectations of pain reduction after treatments were high [28]. The contrast to our results might be due to the review in principal concerned interventions delivered in specialist care while the informants in our study were asked about their expectations towards a non-pharmacological intervention delivered in primary care. Nevertheless, the distinction between ideal and predicted expectations towards outcomes of pain treatment described in the review may be useful for understanding the informants’ expectations to the current interventions. Alternatively, it might be that the informants have not experienced substantial or long-lasting improvement in pain level previously and therefore do not consider it realistic to achieve pain relief. If so, that would be in line with patients with extensive healthcare experience not expecting interventions necessarily to alleviate their symptoms [32].

In line with existing knowledge [29], we found that hope could be seen as a driving force towards trying new interventions or services as it was seen as essential for maintaining motivation to do activities experienced as beneficial. The role of hope for motivation to self-care implies that service providers should be aware of and help to maintain patients’ hopes. A challenge for the health service, though, is how to support hope without creating unrealistic expectations [29] or despair [41], but rather contributing to a sustained hope that would promote self-care.

### Available services when needed

Although there were similarities, some of the expectations towards participation in the easily accessible interventions were in contrast to expectations identified in studies on multimodal and more comprehensive pain programmes [13, 30–32]. Where these studies found that participants had expectations towards health providers’ competency [30], learning specific coping techniques [13], and on outcomes related to diagnostics and causes of pain [31, 32], that was not prominent in our study. A possible reason for the difference might be that what the informants’ perceived primary care could offer them was different from what they sought in specialist health services. Another reason might be that the easily accessible interventions addressed chronic pain regardless of cause or underlying disease, which were different from studies addressing specific pain conditions [30–32]. Professional education and interdisciplinary treatments are pointed to as important in pain treatment [26]. The informants in this study, however, also emphasised the importance of social support comprising both peers and professionals, as part of their self-care. Notably, the informants saw it as more important that the services were available for them whenever they needed help to manage their pain, than who provided the services or their location.

Having access to services when they recognised a need for support was described as important for the informants’ maintenance of self-care. More generally, participating in interventions provided by healthcare services was seen by some as an act of self-care, where they did something active to manage their health. This is similar to Wagner et al.’s description of the patient being the pilot, where the role of the healthcare system is to ensure that the pilots are skilled and capable of getting to their destinations [27]. For health service, though, this poses a challenge of being responsive to the patients’ needs when they arise. Hence, strengthening the patient’s role as informed and activated requires that service providers are given the leeway to support patients’ use of healthcare whenever they need it. However, according to some of the informants this was not always the situation as they had experienced trouble knowing where to turn for help, and requests for help were rejected or postponed. Their experiences exemplify obstacles in the healthcare system when one tries to manage chronic pain, confirming previous findings that access to health services and resources can be difficult [42–44]. This can explain why the informants saw the easy access to the current interventions as a possible solution for their need for a service that was available whenever they needed support to manage their pain.

### Strengths and limitations

A strength in the study is the novelty in exploration of expectations towards easily accessible pain management interventions in public primary care. To minimize potential biases during the analysis, preliminary results were discussed with an extended research group to expose the data for different views and perspectives. However, there are some noteworthy limitations. The sampling strategy could have led to a selected sample as the informants were only recruited among participants agreeing to participate in a RCT. It is thus possible we have missed expectations from people who did not want to participate in a trial. Similar to recruitment to other self-management interventions, there were more women than there were men in the sample. Nevertheless, our sample included informants of different ages and different lengths of pain duration, and we perceive the sample to mirror the participants in the larger trial. In addition, the interview setting could have affected the interviews since the informants were interviewed right after they had answered the RCT’s questionnaires. However, a review of the interviews showed very few references to the questions, indicating they spoke from experience and not the concepts in the questionnaires.

## Conclusion

The informants’ expectations were not specifically related to the pain level being diminished or alleviated. Their hope for a better everyday life was the main driving force towards trying new interventions and health services. The participants perceived the primary care-delivered pain management interventions to be an opportunity for easy access to peer and professional support. The role of hope for peoples’ motivation to self-care implies that service providers should be aware of and help to maintain patients’ hope for a better everyday life. The importance of social support as part of self-care should be acknowledged when developing interventions targeting chronic pain.

## Abbreviations

GP: General Practitioner
HLC: Healthy Life Centre
RCT: Randomised controlled trial
STC: Systematic text condensation
